# Variance in multiplex suspension array assays: microsphere size variation impact

**DOI:** 10.1186/1742-4682-4-31

**Published:** 2007-08-23

**Authors:** Brian P Hanley, Li Xing, R Holland Cheng

**Affiliations:** 1 Microbiology Graduate Group, University of California, Davis, CA 95616, USA; 2 Department of Molecular and Cellular Biology, University of California, Davis, CA 95616, USA

## Abstract

**Background:**

Luminex suspension microarray assays are in widespread use. There are issues of variability of assay readings using this technology.

**Methods and results:**

Size variation is demonstrated by transmission electron microscopy. Size variations of microspheres are shown to occur in stepwise increments. A strong correspondence between microsphere size distribution and distribution of fluorescent events from assays is shown. An estimate is made of contribution of microsphere size variation to assay variance.

**Conclusion:**

A probable significant cause of variance in suspended microsphere assay results is variation in microsphere diameter. This can potentially be addressed by changes in the manufacturing process. Provision to users of mean size, median size, skew, the number of standard deviations that half the size range represents (sigma multiple), and standard deviation is recommended. Establishing a higher sigma multiple for microsphere production is likely to deliver a significant improvement in precision of raw instrument readings. Further research is recommended on the molecular architecture of microsphere coatings.

## Background

A suspended microarray assay system uses small particles such as microrods or microspheres that contain some method for identifying a set, often termed a classifier. Classifiers are often 2 (or 3 in the cased of the new Luminex 3-D system) fluorophores dedicated to the task of identifying a particle set, but may be transponders or some other method. An assay used to detect an analyte is bound to the surface of a set of identically classified particles, which are generally in the size range 3–15 microns. These particles are added to a liquid containing the analyte. (In systems such as "smart dust", the assay may be distributed in the field to detect analytes and read differently.) The final step in the assay activates a reporter fluorophore that provides a signal. In systems using fluorophores for classification, the reporter fluorophore is distinct, and will have a significant frequency difference from the classification fluorophores. The particles are run through a flow cytometer, which is generally optimized for the specific system used. For each particle in the mixture, the cytometer identifies the classifier together with the fluorescence reading of the reporter fluorophore. Because the particle classifiers are unique for each analyte, it is possible to multiplex the assays together in a test tube. Additionally, multi-well assay plates can be used, and such assays then become a high throughput system.

In the Luminex system analyzed here, fluid with a sample of microspheres flows up through a probe, which has a tip with 5 very fine holes leading to a single channel at the top. The fluid travels through a system of tubing and valves into the flow cell, where (in the current equipment) two lasers are present. One laser stimulates the two marker fluorophores, and the other stimulates the reporter fluorophore. A system of avalanche photodiodes and photomultiplier tube captures and reads the fluorescence from marker and reporter emissions.

## Methods

Samples of uncoated microspheres were vortexed, pipetted on to copper grids (Electron Microscopy Sciences, Hatfield, PA) which were carbon coated in the lab prior to use. Images were made by transmission electron microscopy (TEM) using a JEM-1230 (JEOL Ltd. Tokyo, Japan). In total, 23 images with 194 microspheres were obtained. No staining was used since solid polystyrene spheres of 5 microns or larger have high contrast in vacuum. Microspheres were measured on one axis using Adobe Photoshop 5.5 measurement tool. Numbers were overtyped on each measured microsphere using Photoshop. In these TIFF images, 342 pixels = 20 micron. 1 micron = 14.7 pixels. Pixel measurement error, ±1 pixel. Estimated measurement accuracy, ±0.07 micron.

Archived Luminex assay data was used for samples of high level fluorescence from biotin high value assay controls (Table 1).

**Table 1:** Archival data used for calculation of mean brightness of high fluorescence signal control microspheres. All data is from one instrument to eliminate any inter-instrument calibration issues.

	(FσF) MathType@MTEF@5@5@+=feaafiart1ev1aaatCvAUfKttLearuWrP9MDH5MBPbIqV92AaeXatLxBI9gBaebbnrfifHhDYfgasaacH8akY=wiFfYdH8Gipec8Eeeu0xXdbba9frFj0=OqFfea0dXdd9vqai=hGuQ8kuc9pgc9s8qqaq=dirpe0xb9q8qiLsFr0=vr0=vr0dc8meaabaqaciaacaGaaeqabaqabeGadaaakeaadaqadaqaamaalaaabaGaemOrayeabaacciqcaaSae83WdmhdcqWGgbGraaaakiaawIcacaGLPaaaaaa@3318@	*N*	Weighted average of ratios	Total *N*
Dataset 1	6.12	50	7.40	231
Dataset 2	4.34	30		
Dataset 3	12.80	32		
Dataset 4	7.41	23		
Dataset 5	7.23	96		

## Results

**Figure 1:**
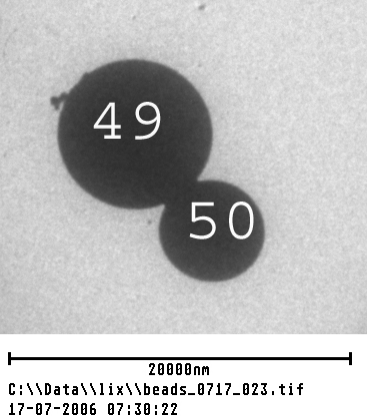
A pair of microspheres of quite different sizes from an image subsection. Numbers 49 and 50 are 9.1 microns and 6.3 microns, respectively, to illustrate degree of size difference.

**Figure 2:**
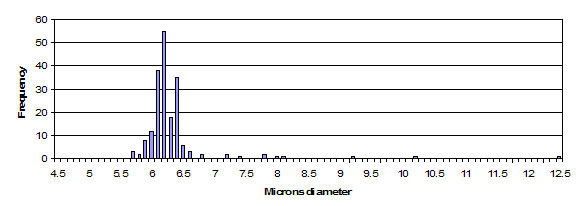
This is a histogram of transmission electron microscopy sample of microsphere sizes for the study, where N (number of measurements) = 194. Compare with Figure 3 to see similarity of distribution, for width of distribution and high side outliers.

Figure 1 shows a sample micrograph subsection that illustrates size differences. The standard deviation for the TEM sample of 194 microspheres was 0.66 microns around a mean of 6.29 microns and a median of 6.14 microns. Microsphere sizes ranged from 5.6 to 12.5 microns diameter. This sample of 194 microspheres had a 0% chance of presenting the results shown (Figure 2) if the true mean of the population was 5.6 microns and the standard deviation was the 0.66 microns determined by TEM.

## Discussion

### Heuristics based estimate of probable microsphere size variance

Luminex supplied a specification for the microspheres of a diameter of 5.4–5.8 microns with a mean of 5.6 n. This is a range of 0.4 microns. The specification was used as a guide to the possible range of diameters for this heuristics based calculation. The specification lacks a sigma multiple.

In manufacturing, a standard high level target for tolerances (acceptable variation in measurement for a dimension of a part) is that 6*σ *(six-sigma) is inside the tolerance [1]. (In this context, *σ *and *s *are generally used interchangeably to mean standard deviation.) A 6*σ *specification means that 1 in 10^8 ^items will fall outside of the specified tolerance. (Motorola defines 'six-sigma' for such processes as ≈±4.5 *σ*, because they assume manufacturing process mean drift of 1.5 *σ *between machine adjustments.) Six-sigma is used here as a 'best case' that can reasonably be expected for the standard deviation in microsphere sizes. Since a normal distribution is two-tailed from its center, one *σ *in a direction is 1/12 of the specified full tolerance range of 0.4 microns. Six-sigma is considered the modern target for manufacturing of electronics and precision parts. However, some industrial processes are run at higher than six-sigma.

Using 6*σ *as the target, the supplied range of 0.4 microns/12 = 0.033 microns as the standard deviation if 6*σ *were the specification. This establishes a reasonable low boundary for a heuristic estimate.

Similarly, in older school manufacturing (which is still used for certain types of parts) good practice for tolerances is to hold ±3*σ *(3 sigma). This means that roughly three items per 1,000 will be out of tolerance specification. Using 3*σ *as the tolerance, it would be expected that the *σ *on microsphere size is 0.4 microns/6 = 0.066 microns as the standard deviation. This will be the middle of our heuristic range estimate.

Less controlled manufacturing processes have higher reject rates, in the order of 1% to 40% failure to hold tolerance. They will hold somewhere around 1 to 2 *σ *of manufactured product within a specified tolerance due to intrinsic or extrinsic factors. Thus, 2*σ *was used as the high boundary. This corresponds to 5% failure to hold tolerance. Thus, using 2*σ *as the specification, 0.4 microns/4 = 0.1 micron as the standard deviation. This is the high estimate.

Low *σ *estimate = 0.033 micron

Middle *σ *estimate = 0.066 micron

High *σ *estimate = 0.01 micron

### Estimate of size based on fluorescent intensity

This experimentally-based estimate first examined the probability that individual microspheres vary significantly in brightness because of random variation in the number of antigens or antibodies bound per microsphere. However, since the sample size (i.e. antigen or antibodies bound) per microsphere is on the order of 10^5 ^to 10^7 ^[2], differences in percentage of analyte bound to microspheres as a source of variance should be approximately zero. In cases with very large antigens, the density on the surface of the microspheres may be lower. However, in such cases (e.g. viral particles), multiple antibody binding sites are likely to be available per antigen surface.

An experimentally-based estimate of the standard deviation of microsphere size was developed and termed "combined virtual". This means that the microsphere size variation derived takes all sources of variation into account. The true variation in size may be less depending on what proportion of it is based on microsphere size, and what proportion on electronic/optical system noise. Using the average for the brightest high value control set (Table 1) allows a simple proportion to be established:

Where:

*F *= maximum fluorescent intensity

*σ*_*F *_= standard deviation of maximum fluorescent intensity

*B *= microsphere surface area

*σ*_*B *_= standard deviation of microsphere surface area

FσF=BσB
 MathType@MTEF@5@5@+=feaafiart1ev1aaatCvAUfKttLearuWrP9MDH5MBPbIqV92AaeXatLxBI9gBaebbnrfifHhDYfgasaacH8akY=wiFfYdH8Gipec8Eeeu0xXdbba9frFj0=OqFfea0dXdd9vqai=hGuQ8kuc9pgc9s8qqaq=dirpe0xb9q8qiLsFr0=vr0=vr0dc8meaabaqaciaacaGaaeqabaqabeGadaaakeaadaWcaaqaaiabdAeagbqaaGGaciab=n8aZXGaemOrayeaaOGaeyypa0ZaaSaaaeaacqWGcbGqaeaacqWFdpWCmiabdkeacbaaaaa@35C0@

σB=B(FσF)
 MathType@MTEF@5@5@+=feaafiart1ev1aaatCvAUfKttLearuWrP9MDH5MBPbIqV92AaeXatLxBI9gBaebbnrfifHhDYfgasaacH8akY=wiFfYdH8Gipec8Eeeu0xXdbba9frFj0=OqFfea0dXdd9vqai=hGuQ8kuc9pgc9s8qqaq=dirpe0xb9q8qiLsFr0=vr0=vr0dc8meaabaqaciaacaGaaeqabaqabeGadaaakeaaiiGajaaOcqWFdpWCmiabdkeacPGaeyypa0ZaaSaaaeaacqWGcbGqaeaadaqadaqaamaalaaabaGaemOrayeabaqcaaQae83WdmhdcqWGgbGraaaakiaawIcacaGLPaaaaaaaaa@38A5@

The data for this proportion were a set of values taken from archived Luminex assays. All assays had a high value biotin control microsphere (which serves as a fully occupied, therefore high fluorescent signal, assay) as hown in Table 1.

Table 1 was developed to fill in the equation for calculating the standard deviation in microsphere diameter for Luminex microspheres. It represents 231 well readings, obtaining (FσF)
 MathType@MTEF@5@5@+=feaafiart1ev1aaatCvAUfKttLearuWrP9MDH5MBPbIqV92AaeXatLxBI9gBaebbnrfifHhDYfgasaacH8akY=wiFfYdH8Gipec8Eeeu0xXdbba9frFj0=OqFfea0dXdd9vqai=hGuQ8kuc9pgc9s8qqaq=dirpe0xb9q8qiLsFr0=vr0=vr0dc8meaabaqaciaacaGaaeqabaqabeGadaaakeaadaqadaqaamaalaaabaGaemOrayeabaacciqcaaSae83WdmhdcqWGgbGraaaakiaawIcacaGLPaaaaaa@3318@ = 7.40. From the Luminex specification diameter = 5.6 microns, so *B *= 4*π*r^2 ^= 4*π*(5.6/2)^2 ^= 98.52 square microns. Substituting these values into equation 2 gives:

*σ*_*B *_= 98.52/7.40 = 13.31 square microns

Thus, the standard deviation of microsphere area corresponds to a sphere of 13.31 microns in area. Such a sphere has a diameter of 213.314π
 MathType@MTEF@5@5@+=feaafiart1ev1aaatCvAUfKttLearuWrP9MDH5MBPbIqV92AaeXatLxBI9gBaebbnrfifHhDYfgasaacH8akY=wiFfYdH8Gipec8Eeeu0xXdbba9frFj0=OqFfea0dXdd9vqai=hGuQ8kuc9pgc9s8qqaq=dirpe0xb9q8qiLsFr0=vr0=vr0dc8meaabaqaciaacaGaaeqabaqabeGadaaakeaacqaIYaGmdaGcaaqaamaalaaabaGaeGymaeJaeG4mamJaeiOla4IaeG4mamJaeGymaedabaGaeGinaqdcciGae8hWdahaaaWcbeaaaaa@352F@ = 2.06 microns.

By this experimentally-derived method, the microsphere combined virtual size standard deviation (i.e from all sources) ≈ 2.06 microns. This combined estimate of microsphere size *σ *should be considerably larger than the true microsphere size variation, since no correction has been made for any other sources of variance. This estimate falls far outside the heuristics based estimate of the previous subsection.

### Transmission electron microscopy based size estimate

The first author's manufacturing experience and instructional literature on quality control [3] indicate that the true mean for any manufactured part varies from lot to lot, between machines used for manufacturing; so does the standard deviation. In addition, preliminary indications from TEM (data not shown) suggested that coated microspheres that have been frozen and thawed repeatedly may show greater variance than uncoated microspheres from this sample.

**Figure 3:**
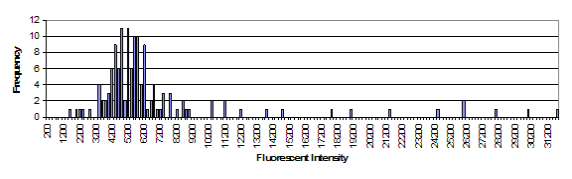
Histogram of a representative sample of events for one classifier from an event set. Classifier shown is region 97, N = 136. This histogram is based on fluorescent intensity of the reporter fluorophore. Compare with Figure 2.

A histogram of the measurement data extracted from the TEM images (Figure 2) shows a distribution quite similar to the distributions of fluorescent intensity as exampled in Figure 3. The standard deviation of 0.66 micron for the TEM sample is over 6 times the calculated standard deviation for a 2-sigma manufacturing [1] process. The sigma multiple has significant impact on assay precision and is a primary focus of discussion.

Microspheres measured by TEM had a mean diameter at or above the published mean of 5.6. That the microspheres mean diameter of 6.14 microns was different from the published specification of 5.6 microns is not inherently a problem. If true mean diameter of microspheres, standard deviation and skew were published by lot, then algorithms could correct them relative to each other. Additionally, where standard curves are used to determine concentrations, true microsphere diameter is automatically adjusted for. Where mean diameter becomes a concern is when assays are used with cutoff values, for example to make a diagnosis. However, size variation will always impact precision.

### Optimal trimming of data could be based on distribution characteristics

Examining microsphere size distribution again (Figure 2), the primary distribution is seen to occur between 5 and 7 microns. Trimming the high end skew above 7 microns removes approximately 5% of the data points in this sample (10 microspheres). This optimized trimming of data results in a standard deviation of 0.18 microns, a significant improvement over 0.66 microns. The improvement in variance is 0.6620.182
 MathType@MTEF@5@5@+=feaafiart1ev1aaatCvAUfKttLearuWrP9MDH5MBPbIqV92AaeXatLxBI9gBaebbnrfifHhDYfgasaacH8akY=wiFfYdH8Gipec8Eeeu0xXdbba9frFj0=OqFfea0dXdd9vqai=hGuQ8kuc9pgc9s8qqaq=dirpe0xb9q8qiLsFr0=vr0=vr0dc8meaabaqaciaacaGaaeqabaqabeGadaaakeaadaWcaaqaaiabicdaWiabc6caUiabiAda2iabiAda2maaCaaaleqabaGaeGOmaidaaaGcbaGaeGimaaJaeiOla4IaeGymaeJaeGioaGZaaWbaaSqabeaacqaIYaGmaaaaaaaa@368A@ = 13.45 times.

Such an improvement in variance is probably within reach of an improved algorithm that uses as its input the true size distribution of the microspheres being read. Such a distribution could be developed by quality control sampling of microsphere size, which could be automated. This proposal's relationship to trimmed values is unknown.

### How much of the variance in fluorescent intensity is probably due to the microspheres?

The ratio of the standard deviation of microsphere size distribution over the standard deviation of luminance 'combined virtual' is 0.662.06
 MathType@MTEF@5@5@+=feaafiart1ev1aaatCvAUfKttLearuWrP9MDH5MBPbIqV92AaeXatLxBI9gBaebbnrfifHhDYfgasaacH8akY=wiFfYdH8Gipec8Eeeu0xXdbba9frFj0=OqFfea0dXdd9vqai=hGuQ8kuc9pgc9s8qqaq=dirpe0xb9q8qiLsFr0=vr0=vr0dc8meaabaqaciaacaGaaeqabaqabeGadaaakeaadaWcaaqaaiabicdaWiabc6caUiabiAda2iabiAda2aqaaiabikdaYiabc6caUiabicdaWiabiAda2aaaaaa@3440@ = 0.32. By this measure, roughly 32% of the difference seen in fluorescent intensity readings could be due to variation in microsphere size. Using the formal definition of variance, 0.6622.062
 MathType@MTEF@5@5@+=feaafiart1ev1aaatCvAUfKttLearuWrP9MDH5MBPbIqV92AaeXatLxBI9gBaebbnrfifHhDYfgasaacH8akY=wiFfYdH8Gipec8Eeeu0xXdbba9frFj0=OqFfea0dXdd9vqai=hGuQ8kuc9pgc9s8qqaq=dirpe0xb9q8qiLsFr0=vr0=vr0dc8meaabaqaciaacaGaaeqabaqabeGadaaakeaadaWcaaqaaiabicdaWiabc6caUiabiAda2iabiAda2maaCaaaleqabaGaeGOmaidaaaGcbaGaeGOmaiJaeiOla4IaeGimaaJaeGOnayZaaWbaaSqabeaacqaIYaGmaaaaaaaa@3688@ = 0.10 or 10% of variance probably due to size. Note that total variance (where variance is denoted by *V*) is composed of:

*V*_*Microsphere size *_+ *V*_*Opto-electronic *_+ *V*_*Bench *_+ *V*_*Other*_

Thus, by this estimate, the remaining 68 – 90% of variance is composed of all other sources, with opto-electronic system variance being one component of this remainder. Other experiments (not shown) suggest that bench variance is a quite significant source. Taken together, this suggests that microsphere size is a reasonable target for improvement of assay precision.

### Probability of selecting N microspheres significantly outside the mean size

We now discuss a model for the probability of drawing a number of microspheres, *N*, from a well, all of which are above (or by extension, below) the mean diameter of spheres specified in Luminex literature at 5.6 microns using 5.4 – 5.8 microns as the range.

**Figure 4:**
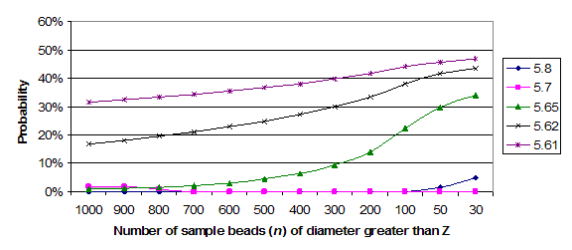
Graphs of the probability of *N *microspheres above size Z when microsphere size sigma = 0.66 microns.

Figure 4 shows the probability that *N *microspheres in a sample would have a diameter greater than the *Z *value shown, given the microsphere standard deviation of 0.66 microns determined by TEM. The values of *N *shown were selected because they correspond to the published range [4]. (Although the graph is continuous, such events happen in a discrete distribution, the quantum being a single microsphere.) This topic could be developed further in a mathematical summation to map the 'phase space' to clarify its implications. However, the relevant point should be clear from the graph: the mean fluorescent intensity for an identical homogenous sample can vary significantly for purely stochastic reasons. The basis of a major source of stochastic variance is the distribution of microsphere sizes.

### Relationship of size to brightness

**Figure 5:**
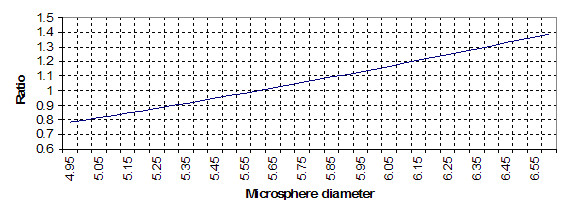
Graph of the ratio of fluorescent intensity to true normal by bead size. A ratio of 1 is for a microsphere sample equal to the specified mean microsphere diameter. This shows the relationship of microsphere diameter to brightness of signal, illustrating the correspondence of brightness as an r^2 ^relation.

It is useful to have as high a sigma multiple as possible for a set of manufactured microspheres. Figure 5 shows the problem inherent in use of microspheres that do not have tight control on microsphere size. Although microsphere surface area varies with the square of the radius, this region is so small it is for practical purposes linear.

If the entire microsphere sample is above the value given, it will present a distribution of microspheres displaying a higher fluorescent intensity for the same sample. This fluorescent range will be variable, and may be skewed. However, for the sake of simplicity, the center of the distribution will be assumed to be at least 1.3 × *σ *microns above the value shown. This must be assumed because if all of the microspheres in the sample are above the size specified, the sample will still have a normal distribution of sizes with a mean higher than that of the smallest microsphere. The assumption that the mean of the sample is only 1.3 standard deviations from the edge of its distribution is quite conservative; it can easily be higher.

As an example, a relatively common occurrence for a 100 microsphere count sample is an approximately 22% probability that all microspheres are larger than 5.65 microns (Figure 4). (And by extension, in a balanced normal distribution, 22% are smaller than 5.0 microns.) This would be a microsphere set centered at 5.65 ± (~1.3 × 0.66) microns, or 5.0 – 6.25 microns. (0.66 is the standard deviation from the TEM results.) Using the proposed model, a sample centered exactly on 5.6 microns would have a fluorescent intensity ratio of 1.0 (figure 5), while a sample at 6.26 microns mean diameter would have fluorescent intensity ratio of 1.26 to the nominal mean value, an increase of 26% in fluorescent intensity for the specific microsphere set for the well. Similarly, the same probability exists that all diameters in the sample are or 26% lower. This indicates that roughly 50% (i.e. ≈27% × 2) of results would occupy a range of 26% higher or lower than they should be, from one well to the next.

### Comparison of worked example with results

When averaged over an entire plate of 96 wells with a 25-plex assay in each well (data not shown), the mean high side variation from the mean was +59%. The mean low side variation is -56% from the sample mean. These figures were derived from trimmed mean data. This indicates, as expected, that the total summation of variation has an upper and lower bound greater and lower, respectively than the variation in the preceding proposed model (±26%). This is in reasonable agreement with the example workup in relationship of size to brightness above.

### Microsphere sizes in TEM data display an exponential regression for outlier sizing

**Figure 6:**
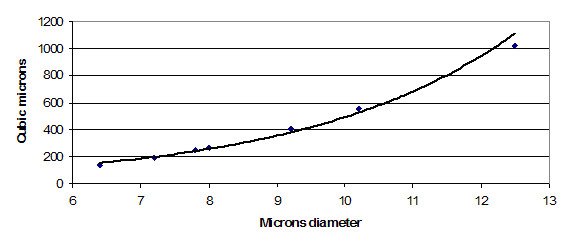
Regression on microsphere volume above 7 microns diameter. The spacing of data points along this fitted curve indicates that microspheres are enlarged by stepwise increments. This suggests that the reason for variation in size is a problem of detachment from injector nozzles.

Figure 6 shows sizes expressed as volume of microspheres for all existing data points above 7 microns, using the volume of the mean 6.4 micron diameter as the first reference datapoint. A pattern such as this, with rough doubling of volume between occurrences, most likely indicates a regular manufacturing process issue that can be corrected.

## Conclusion

The overall conclusion is that a probable significant cause of variance in suspended microsphere assay results is the variation in microsphere diameter. Lowering the standard deviation of microsphere size and improving the sigma multiple for lot quality control appears likely to be an area for improvement. Doing so will lower the amount of stochastic variance of this type of assay system, and improve the precision of these assays. Provision of accurate mean size, median size, skew and sigma multiple for the size range would be useful to users. Additionally, there is a need for more research into the details of the microparticles used in suspended microarray assay systems, how surfaces are coated and the molecular architecture resulting from various coating protocols.

## Competing interests

The author(s) declare that they have no competing interests.

## Appendix

### Approximate density of antibody per microsphere

It was decided to provide this workup as an appendix because there is no other published estimate of binding sites that describes how the estimate was done.

According to Luminex, carboxylation sites are present on the polystyrene molecules prior to forming the microspheres [5]. Consequently, an overabundance and even distribution of binding sites should be expected.

At its base, each antibody (Ab) is 52 angstroms in diameter, 150 angstroms at the top and 160 angstroms long.

160 angstroms = 0.016 microns, i.e. 0.006 of the radius of a microsphere.

50 angstroms is 0.005 microns. A circle 160 angstroms in diameter is assigned as the region on the surface of a microsphere for each attachment. This seems a reasonable value for now, since the length of each Ab is such a tiny percentage of the radius of the sphere. There will be no significant difference in size on the surface from the base to the ends of the Y in the antibody in terms of the radian angle needed. It is set to 160 to give 5 angstroms minimum clearance on a side.

Surface area of a sphere is 4*π*r^2 ^which for the Luminex microspheres ~4π * 2.8^2 ^= 98.52 microns^2^. 1 micron = 10^5 ^angstroms. 1 micron^2 ^= 10^10 ^angstroms^2^.

This Ab circle occupies an area of *π*r^2 ^for the Ab. That is *π*0.008^2 ^= 0.00020106 microns^2^. Adding the size of the triangular section required to tile the surface, this becomes 0.000222 microns^2^

98.52 microns^2^/2.22 10^-4 ^= 4.4438 × 10^5^

*Maximum probable number of Ab per microsphere ~ 4.8 × 10^5^.*

However, if one assumes that the Abs can crowd somewhat, by removing the triangular section, a larger number of roughly 4.8 × 10^7 ^is obtained. This larger number is quite reasonable since the geometry of antibodies allows it.

±2 to 4 orders of magnitude isabout as good as can be expected by this method given the unknowns.

*Maximum total is roughly 4.8 × 10^5^/6.02 × 10^23 ^= 7.37 × 10^-19 ^moles of detection per microsphere.*

A figure supplied by Luminex is 6.7 × 10^5 ^binding sites per microsphere[2]. In discussion with Luminex chief of R&D, the methods used are roughly equivalent to those used in this appendix, and shows good agreement.
